# Regional and Seasonal Dynamics of Leptospirosis in Ukraine, 2023–2025

**DOI:** 10.3390/pathogens15070759

**Published:** 2026-07-20

**Authors:** Pavlo Petakh, Iryna Halabitska, Oleh Lushchak, Oleksandr Kamyshnyi

**Affiliations:** 1 Department of Biochemistry and Pharmacology, Uzhhorod National University, 88000 Uzhhorod, Ukraine; 2 Kyiv School of Economics, 03113 Kyiv, Ukraine; 3 Department of Therapy and Family Medicine, I. Horbachevsky Ternopil National Medical University, 46001 Ternopil, Ukraine; halabitska@tdmu.edu.ua; 4 Department of Microbiology, Virology, and Immunology, I. Horbachevsky Ternopil National Medical University, 46001 Ternopil, Ukraine

**Keywords:** leptospirosis, Ukraine, surveillance, seasonality, climate, river network density, forest cover, random forest

## Abstract

**Background**: Ukraine has substantial regional differences in climate, landscape, forest cover, and river networks, which may influence leptospirosis transmission. However, recent monthly oblast-level variation in leptospirosis incidence has not been systematically described. We used newly available surveillance data for 2023–2025 to assess seasonal and regional patterns of leptospirosis and their associations with weather, climatic zone, forest cover, and river network density. **Methods**: We analyzed surveillance data from 23 Ukrainian oblasts. Incidence was assessed by month, oblast, and climatic zone. Weather data were aggregated monthly; forest cover and river network density were included as predictors. Associations were assessed using correlation, cross-correlation, and Random Forest ML models. **Results**: Incidence showed a clear seasonal increase, reaching its highest levels in late summer and autumn, approximately one to three months after seasonal peaks in temperature and precipitation. The highest incidence was observed in Zakarpattia, Chernihiv, and Ternopil oblasts, while incidence by climatic zone was highest in the Carpathian group and lowest in the Steppe zone. Among weather variables, average temperature showed the clearest delayed association with leptospirosis incidence. River network density was the leading ecological predictor in the adjusted models. The positive unadjusted association between forest cover and incidence became negative after adjustment for river network density, suggesting that these variables captured overlapping ecological characteristics. In the Random Forest analysis, river network density was the top-ranked predictor, and the best-performing model achieved an AUC of 0.795. **Conclusions**: Leptospirosis incidence in Ukraine varied substantially by season and region. Delayed temperature effects, river network density, and forest cover were associated with regional risk. Monthly oblast-level surveillance with climatic and ecological data may help monitor leptospirosis risk, but war-related disruption to diagnosis and reporting should be considered.

## 1. Introduction

Leptospirosis is an important zoonotic infectious disease in Ukraine with substantial public health relevance, especially during the current period of war [1,2,3]. It is caused by pathogenic *Leptospira* spp. and is transmitted through direct or indirect contact with urine from infected animals, particularly rodents, or with contaminated water and soil [4,5,6]. The clinical spectrum of leptospirosis ranges from mild febrile illness to severe and potentially fatal disease involving renal, hepatic, pulmonary, or hemorrhagic complications [7,8].

Multiple interacting factors influence leptospirosis transmission, including animal reservoirs, socio-economic conditions, occupational exposure, human behaviour, geographical factors such as river networks, forest coverage, and land use, and weather conditions [9,10]. In Ukraine, occupational exposure has become especially important in the context of war, as military personnel may work in trenches, wetlands, agricultural areas, or other field environments where contact with soil, mud, surface water, and rodents is common [11,12]. Weather-related factors, including temperature, precipitation, flooding, and soil moisture, can influence transmission risk and be associated with seasonal increases or outbreaks of leptospirosis [13,14,15].

Previous global burden estimates reported that leptospirosis causes approximately 1.03 million cases and 58,900 deaths annually worldwide [16]. In the EU/EEA, a recent analysis by Beauté et al. reported 12,180 confirmed leptospirosis cases during 2010–2021, with an average annual reported incidence of 0.24 cases per 100,000 population [17]. The largest number of cases was reported in France, with 6058 cases. Although Ukraine was not included in this analysis, national surveillance data for the same period recorded 4431 leptospirosis cases, with an average annual incidence of 0.72 cases per 100,000 population [1].

The real burden of leptospirosis is probably higher than official numbers suggest [16,18]. Early symptoms, such as fever, myalgia, headache, and gastrointestinal upset, are not specific and can easily be confused with other febrile illnesses [19,20]. Without laboratory confirmation, some cases may be missed [21]. This is especially relevant during the war in Ukraine, when access to health care and laboratory testing has been disrupted in some regions and population displacement may have affected routine reporting [22,23].

Ukraine has marked regional differences in climate, relief, river density, forest coverage, and land use, which may create different local conditions for leptospirosis transmission. In our previous analysis, regions with denser river networks showed higher leptospirosis incidence. However, that study was limited by the structure of surveillance data available for 2018–2023, as monthly region-level data were not accessible [24]. Recent changes in how surveillance data are stored now make it possible to analyze leptospirosis by month and oblast, allowing a more detailed assessment of regional, seasonal, and weather-related variation.

This study therefore used newly available monthly oblast-level surveillance data to examine leptospirosis in Ukraine during 2023–2025. The main objectives were to describe regional and seasonal dynamics, assess associations with weather-related and regional factors, and test whether these variables could be used for exploratory risk stratification.

## 2. Materials and Methods

### 2.1. Study Design, Setting, and Data Sources

This retrospective observational study was based on national and regional surveillance data on leptospirosis in Ukraine during 2023–2025. The study aimed to describe regional and seasonal variation in reported leptospirosis incidence and to assess its association with selected weather-related and geographical factors. Ukraine was analyzed at the oblast level, using administrative regions as the main spatial units of analysis.

Monthly data on reported leptospirosis cases in Ukraine were obtained from the Public Health Centre of Ukraine (https://phc.org.ua/, accessed on 1 March 2026). Additional regional characteristics were collected, including climatic-zone classification and forest coverage. Climatic-zone classification was performed at the oblast level. Each oblast was assigned to one of four climatic-zone groups: Polissia/forest zone, forest-steppe, steppe, or Carpathian mountain zone. The Polissia/forest zone included Volyn, Rivne, Zhytomyr, and Chernihiv oblasts; the Forest-steppe included Kyiv Oblast, Kyiv City, Sumy, Poltava, Cherkasy, Vinnytsia, Khmelnytskyi, Ternopil, and Lviv oblasts; the Steppe included Kirovohrad, Kharkiv, Dnipropetrovsk, Zaporizhzhia, Mykolaiv, Odesa, and Kherson oblasts; and the Carpathian mountain climate group included Zakarpattia, Ivano-Frankivsk, and Chernivtsi oblasts.

Weather data were obtained from publicly available meteorological datasets (https://meteopost.com/, accessed on 1 March 2026) and aggregated by oblast and month. The main meteorological variables included average temperature, minimum temperature, maximum temperature, precipitation, humidity, and wind speed. Forest coverage (%) was used as an oblast-level environmental variable. Data were obtained from the State Forest Resources Agency of Ukraine based on the 2011 forest inventory. River network density (km of rivers per km^2^ of oblast area) was included as a complementary hydrological predictor and was derived from the State Agency of Water Resources of Ukraine.

### 2.2. Surveillance System in Ukraine and Case Confirmation

Leptospirosis is subject to mandatory notification in Ukraine. Suspected and confirmed cases are reported through the national infectious disease surveillance system according to Ministry of Health regulations. Case identification is based on clinical presentation, epidemiological history, and laboratory confirmation. Clinical criteria include acute febrile illness with compatible symptoms such as chills, headache, myalgia, conjunctival hyperemia, jaundice, hemorrhagic manifestations, renal involvement, meningitis, myocarditis, or respiratory manifestations. Laboratory confirmation was performed mainly using the microscopic agglutination test (MAT) [25], with PCR used in selected cases. Blood samples were tested in Especially Dangerous Infections laboratories according to national methodological recommendations. Paired sera were initially screened at dilutions of 1:5 and 1:50; if agglutination was observed, serial dilutions were performed at 1:10, 1:100, 1:200, and higher. The endpoint titre was defined as the highest serum dilution showing approximately 50% agglutination compared with a control culture diluted 1:2 in phosphate-buffered saline. Results were reported as reciprocal endpoint titres. Titres of ≥1:100, together with compatible clinical and epidemiological data, were considered indicative of infection, while a fourfold rise in titre between paired samples was considered strong evidence of acute infection. The MAT panel comprised 14 reference *Leptospira* serovars: Copenhageni, Grippotyphosa, Canicola, Pomona, Tarassovi, Kabura, Polonica, Poi, Autumnalis, Bratislava, Djatzi, Ballum, Pyrogenes, and Cynopteri. PCR, based on the *lipL32* gene, was used in selected cases, including fatal cases or when tissue samples were available.

### 2.3. Statistical Analysis

Descriptive statistics were used to summarize leptospirosis incidence and predictor variables. Continuous variables were presented as means with standard deviations or medians with interquartile ranges, depending on their distribution. Categorical variables were presented as counts and percentages.

Regional and seasonal patterns were assessed by comparing incidence across oblasts, months, seasons, and the four climatic zones (Polissia, Forest-steppe, Steppe, and Carpathian). Choropleth maps, time-series plots, and bar charts were used to visualize spatial and temporal variation in leptospirosis incidence. Spearman and Pearson correlation coefficients were used to evaluate associations between leptospirosis incidence and weather variables, climatic zone, forest cover ratio, and river network density. Cross-correlation analysis (Pearson r, lags 0–6 months, weather leading incidence) was performed separately for precipitation and average temperature, at the national level, by climatic zone, and for each of the 23 oblasts individually.

Partial Spearman correlations were computed to assess the independent contributions of forest cover ratio and river network density to leptospirosis incidence after controlling for lag-1 and lag-2 weather variables and, additionally, for the other ecological predictor. Because monthly oblast-level case counts showed substantial overdispersion, a Negative Binomial generalized additive model was fitted. The monthly number of reported cases was used as the outcome, with the logarithm of the oblast population included as an offset. Two-month-lagged average temperature, one-month-lagged precipitation, forest cover ratio, river network density, and climatic zone were included as predictors. Continuous predictors were modelled using regression splines to allow for nonlinear associations, whereas climatic zone was included as a categorical fixed effect. The Negative Binomial model was compared with an equivalent Poisson model using the Akaike information criterion and a likelihood-ratio test. A hierarchical Poisson-lognormal model was also fitted to account for repeated observations within oblasts, year-to-year variation, and residual overdispersion. The model included random intercepts for oblast and year and an observation-level random effect, with the logarithm of oblast population included as an offset. Standardized environmental variables and climatic zone were included as fixed effects.

Random Forest classification models were fitted to assess whether routinely available seasonal, weather, and ecological variables could support oblast-month risk stratification. The binary outcome was defined as moderate/high versus low risk using the median incidence among non-zero observations as the threshold. Different predictor configurations, including month of the year, lag-1 and lag-2 weather variables, climatic zone, forest cover ratio, and river network density, were evaluated under three cross-validation schemes: random 5-fold cross-validation, leave-one-climatic-zone-out, and leave-one-oblast-out. Model performance was assessed using the area under the receiver operating characteristic curve, sensitivity, specificity, balanced accuracy, positive predictive value, and negative predictive value.

All statistical analyses and visualizations were performed using R version 4.5.3 (R Foundation for Statistical Computing, Vienna, Austria). Data processing, descriptive analyses, correlation and cross-correlation analyses, count-data modelling, machine-learning analyses, and geospatial visualizations were conducted using tidyverse, stats, forecast, caret, mgcv, randomForest, pROC, ggplot2, and sf.

## 3. Results

### 3.1. Seasonal Dynamics of Leptospirosis Incidence

**Figure 1:**
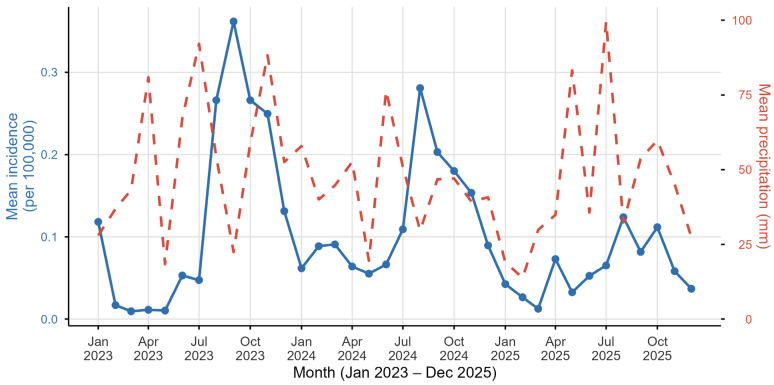
National monthly leptospirosis incidence and precipitation in Ukraine, January 2023–December 2025. The blue line shows the mean monthly leptospirosis incidence per 100,000 population across 23 oblasts, while the red dashed line shows the mean monthly precipitation.

During the study period, 1065 laboratory-confirmed leptospirosis cases were reported across the 23 analyzed oblasts of Ukraine: 433 cases in 2023, 409 cases in 2024, and 223 cases in 2025. Across all 23 oblasts and three years, the mean monthly leptospirosis incidence was 0.103 cases per 100,000 population. Incidence showed clear seasonality, rising from low levels in February–May (0.03–0.04 per 100,000) to a peak in August–October (0.19–0.22 per 100,000). This peak lagged behind the seasonal peak in mean precipitation in July by roughly 1–3 months and behind the seasonal peak in temperature in July–August by a similar interval, consistent with the time needed for *Leptospira* to proliferate in warm, wet environments and for human exposure, incubation, and reporting to occur (Figure 1).

**Figure 2:**
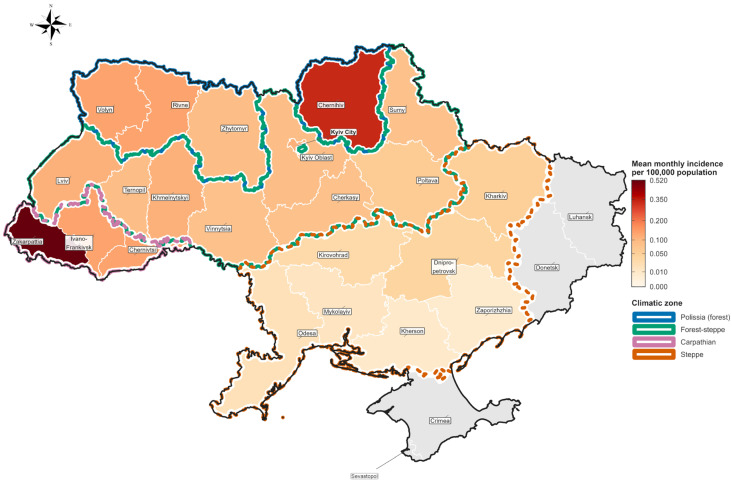
Spatial distribution of mean monthly leptospirosis incidence across Ukrainian oblasts, 2023–2025. Oblast fill colour indicates mean monthly leptospirosis incidence per 100,000 population, with darker red shades corresponding to higher incidence. Coloured outlines indicate climatic-zone classification: Polissia, forest-steppe, steppe, and Carpathian zones. Grey areas indicate oblasts not included in the analysis or territories without available data. Administrative boundary data were obtained from GADM version 4.1. The map was created by the authors in R, and no commercial basemap was used.

The highest mean monthly incidence was observed in Zakarpattia Oblast, at 0.52 cases per 100,000 population. This was followed by Chernihiv Oblast, with 0.33 cases per 100,000 population, and Ternopil Oblast, with 0.22 cases per 100,000 population. Volyn and Ivano-Frankivsk oblasts also had relatively high incidence, both reaching approximately 0.13 cases per 100,000 population. Zakarpattia Oblast had the highest mean monthly incidence, at 0.52 cases per 100,000 population, followed by Chernihiv Oblast at 0.33 and Ternopil Oblast at 0.22 cases per 100,000 population. Volyn and Ivano-Frankivsk oblasts also showed relatively elevated incidence, each reaching approximately 0.13 cases per 100,000 population (Figure 2).

**Figure 3:**
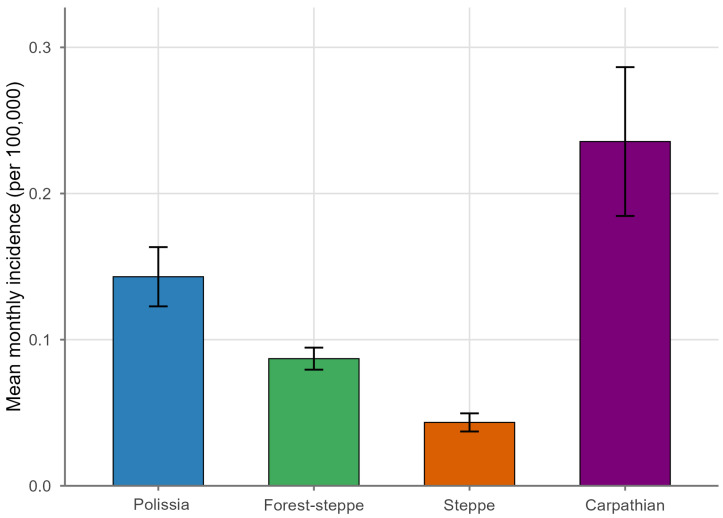
Mean monthly leptospirosis incidence by climate zone, 2023–2025. Oblasts were grouped into four climatic zones: Polissia/forest, forest-steppe, steppe, and Carpathian mountain. Bars show the mean monthly incidence per 100,000 population, and error bars indicate the standard error. The highest incidence was observed in the Carpathian mountain zone, whereas the lowest incidence was observed in the steppe zone.

When oblasts were grouped into four climate-zone categories, leptospirosis incidence differed substantially between zones. The highest mean monthly incidence was observed in the Carpathian mountain climate group, at 0.236 cases per 100,000 population. This was followed by the Polissia (forest zone), with 0.143 cases per 100,000 population. Lower values were observed in the Forest-steppe zone, at 0.087 cases per 100,000 population, and in the Steppe zone, which had the lowest mean monthly incidence, at 0.043 cases per 100,000 population (Figure 3).

### 3.2. River Network Density, Forest Cover, and Leptospirosis Incidence

Two static oblast-level ecological characteristics were evaluated in relation to leptospirosis incidence: forest cover ratio, defined as the percentage of oblast area covered by forest according to the 2011 national inventory, and river network density, defined as kilometres of rivers per square kilometre of oblast area according to the Ukrainian State Water Cadastre. Forest cover ranged from 3.7% to 51.4%, while river network density ranged from 0.15 to 1.25 km/km^2^.

**Figure 4:**
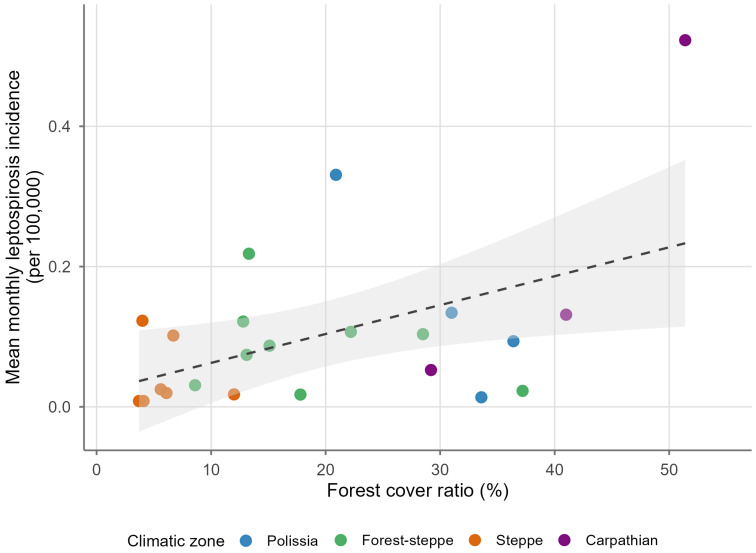
Association between forest cover ratio and mean monthly leptospirosis incidence across Ukrainian oblasts. Each point represents one oblast. The x-axis shows forest cover ratio (% of oblast area under forest), and the y-axis shows mean monthly leptospirosis incidence per 100,000 population during 2023–2025. Points are coloured by climate-zone group.

At the cross-regional level, both ecological variables showed positive associations with mean monthly leptospirosis incidence. River network density showed the stronger association, with a Spearman correlation coefficient of 0.54 (*p* = 0.008), whereas forest cover ratio showed a positive but weaker association (rho = 0.40, *p* = 0.058). In pooled region-month analyses, both variables were significantly associated with leptospirosis incidence (*n* = 782), with a stronger correlation observed for river network density (rho = 0.27, *p* < 0.001) than for forest cover ratio (rho = 0.20, *p* < 0.001) (Figure 4).

Both ecological variables also retained positive partial correlations with log-transformed incidence after adjustment for lagged weather variables. The partial correlation was stronger for river network density (partial rho = +0.28, *p* < 0.001) than for forest cover ratio (partial rho = +0.18, *p* < 0.001). However, after simultaneous adjustment for lagged weather variables and the other ecological predictor, river network density remained independently and positively associated with leptospirosis incidence (partial rho = +0.23, *p* < 0.001), whereas forest cover ratio showed a weak inverse partial association (partial rho = −0.09, *p* = 0.011). This suggests that the apparent positive association between forest cover and leptospirosis incidence may be partly explained by its strong collinearity with river network density (rho = 0.89).

### 3.3. Weather–Incidence Associations

Cross-correlation analysis showed that average temperature was the climatic variable most consistently associated with monthly leptospirosis incidence, particularly at lags of one to three months. At the national level, the strongest correlation was observed for average temperature lagged by two months (Spearman’s rho = 0.34). In oblast-level analyses, positive temperature–incidence correlations peaking at one- to three-month lags were observed in most regions, and these correlations frequently exceeded the approximate 95% significance threshold for the regional time series. A similar pattern was observed in the climatic-zone analysis, with the strongest associations detected in the forest-steppe zone (rho = 0.73 at lag 2) and the Carpathian zone (rho = 0.55 at lag 2).

Precipitation showed a weaker association with leptospirosis incidence. In most oblasts, lagged precipitation was not significantly correlated with incidence, although positive associations were observed in Zakarpattia, Ivano-Frankivsk, Khmelnytskyi, and Kyiv City. At the climatic-zone level, precipitation was significantly associated with incidence only in the Carpathian zone, where correlations were observed at lags of two to three months (rho = 0.50–0.51).

### 3.4. Environmental Modelling and Exploratory Risk Stratification

Monthly oblast-level case counts were overdispersed, with a variance-to-mean ratio of 6.7. Therefore, a Negative Binomial distribution was used instead of a Poisson distribution. The Negative Binomial generalized additive model had a lower AIC than the equivalent Poisson model (ΔAIC = 340), and the likelihood-ratio test favoured the Negative Binomial specification (*p* < 0.001).

Two-month-lagged average temperature showed the strongest nonlinear association with incidence (χ^2^ = 157.0, df = 5, *p* < 0.001), with incidence increasing above 10 °C. River network density (χ^2^ = 68.5, df = 4, *p* < 0.001), forest cover ratio (χ^2^ = 69.9, df = 4, *p* < 0.001), and climatic zone (χ^2^ = 49.4, df = 3, *p* < 0.001) were also associated with incidence. Among the evaluated predictors, one-month-lagged precipitation showed the weakest association with incidence (χ^2^ = 9.5, df = 4, *p* = 0.049). 

Forest cover and river network density showed a high correlation (ρ = 0.89). After adjustment, river network density remained positively associated with incidence, whereas the partial effect of forest cover was negative. This result should not be interpreted as a protective effect of forest cover, because the positive unadjusted association was largely explained by its correlation with river network density.

In the hierarchical Poisson-lognormal model, river network density had the largest coefficient (+1.25 on the log-rate scale per standard deviation), followed by two-month-lagged temperature (+0.69). The coefficient for lagged precipitation was +0.12, whereas forest cover had a negative adjusted coefficient (−0.44). The standard deviations of the random effects were 0.58 for oblast, 0.28 for year, and 0.73 for the observation-level effect.

**Figure 5:**
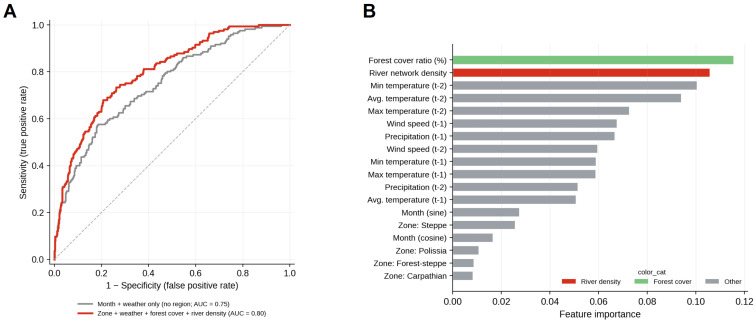
Machine learning-based leptospirosis risk classification and predictor importance. (**A**) Receiver operating characteristic curves for Random Forest classifiers predicting moderate/high leptospirosis risk at the oblast-month level. The grey curve represents the model based on calendar month and weather variables, whereas the red curve represents the model additionally including ecological predictors and climatic-region group. (**B**) River network density (red) and forest cover ratio (green) ranked as the two most important predictors, ahead of average temperature lagged by two months.

The Random Forest classifier achieved an AUC of 0.795 under 5-fold cross-validation, with a sensitivity of 0.588, specificity of 0.855, and balanced accuracy of 0.721. River network density and forest cover were the highest-ranked predictors. Under leave-one-climatic-zone-out and leave-one-oblast-out validation, the AUC decreased to 0.64–0.67 (Figure 5).

## 4. Discussion

This study used newly available monthly oblast-level surveillance data to describe regional and seasonal patterns of leptospirosis in Ukraine during 2023–2025 and to assess their association with weather, climatic zone, forest cover, and river network density. Reported incidence showed clear seasonality, rising from low levels in spring to a peak in late summer and autumn that lagged the seasonal peaks in temperature and precipitation by one to three months. Regional variation was substantial, with more than a 30-fold difference between the highest- and lowest-incidence oblasts, broadly following a climatic-zone gradient from the Carpathian and Polissia/forest zones through the Forest-steppe to the Steppe zone. The count-data models provided a more detailed interpretation of these associations. The Negative Binomial generalized additive model identified two-month-lagged temperature as the strongest meteorological predictor, while river network density was the leading ecological predictor. In the hierarchical Poisson-lognormal model, these associations remained after accounting for differences between oblasts, years, and individual observations. Forest cover and river network density were highly correlated, indicating that they captured partly overlapping ecological characteristics.

The one- to three-month lag between temperature and incidence observed at the national, zonal, and oblast level is consistent with findings from a range of settings, although the precise lag length varies considerably between studies. In Reunion Island, monthly incidence showed significant associations with both cumulated rainfall and mean temperature recorded two months earlier [26], similar to the lag-2 association identified here for the Forest-steppe and Carpathian zones. In Salvador, Brazil, where leptospirosis is highly seasonal and hyperendemic, weekly incidence responded to rainfall and temperature anomalies within one to two weeks [27], reflecting the faster transmission dynamics typical of tropical urban settings. In Fiji, total precipitation over the preceding weeks was identified as the strongest climatic predictor of incidence [28], in contrast to the temperature-dominated pattern observed in the present study. At the other extreme, multi-province analyses in Thailand identified optimal lags of eight to ten months between rainfall or temperature and case counts [29]. This range illustrates that the lag structure linking weather to leptospirosis incidence is highly setting-specific; within it, the one- to three-month temperature lag identified for Ukraine is consistent with a temperate, markedly seasonal transmission pattern, and aligns with recent observations from the Netherlands, where rising temperatures have accompanied an increasing incidence of autochthonous leptospirosis over the past two decades [30].

A lag of this length is compatible with the biology of *Leptospira*, which can persist for weeks to months in warm, moist soil and surface water before sufficient environmental contamination accumulates to produce a detectable rise in human cases [31]. Warmer temperatures may prolong the environmental survival and persistence of pathogenic *Leptospira* and support higher rodent reservoir abundance or activity. In addition, the time required for human exposure, incubation, care-seeking, diagnostic testing, and laboratory confirmation may further delay the interval between favourable environmental conditions and reported case notification [7]. The comparatively weak and spatially heterogeneous association with precipitation observed in this study, in contrast to the more consistent temperature signal, may indicate that leptospirosis transmission in much of Ukraine is less strongly driven by acute flooding events, which are the mechanism most often linked to precipitation in tropical settings [31]. Instead, transmission may depend more on the gradual development of warm and moist environmental conditions that favour the persistence of pathogenic *Leptospira* and support rodent reservoir activity.

Forest cover was positively associated with incidence in the unadjusted analyses. However, forest cover and river network density were highly correlated, and the forest-cover association became negative after adjustment for river network density. This finding does not suggest that forest cover reduces leptospirosis risk. Instead, the two variables appear to represent overlapping landscape and hydrological characteristics, making their separate effects difficult to distinguish. This interpretation is consistent with findings from northern Thailand, where rodent infection was associated with forested landscapes, while human infection was more closely related to proximity to rivers [32]. Previous reviews have also found land use and hydrological conditions as important environmental factors in leptospirosis transmission [9].

The climatic-zone gradient observed in this study, with the highest incidence in the Carpathian and Polissia/forest zones and the lowest incidence in the Steppe zone, places Ukraine within a broader European pattern in which leptospirosis incidence is often higher in more humid, forested, and hydrologically dense regions. Several higher-incidence oblasts identified in the present study, particularly Zakarpattia, had incidence levels exceeding the EU/EEA average of 0.24 cases per 100,000 population reported for 2010–2021 [17]. A recent continent-wide modelling study projected that climate change may expand the geographical and seasonal suitability for leptospirosis transmission in Europe, with northern and central regions historically characterized by cooler climates becoming increasingly suitable under future climate scenarios [15]. If similar processes occur in Ukraine, the climatic and ecological gradients identified here, particularly the roles of temperature, forest cover, and river network density, may become relevant to a wider range of oblasts over time. This reinforces the value of establishing a baseline characterization of regional and seasonal leptospirosis dynamics, as undertaken in the present study.

The exploratory machine-learning results are broadly consistent with the growing literature on climate-informed early warning systems for infectious diseases. In the present study, adding ecological and climatic-zone variables to a model based on calendar month and lagged weather improved discriminative performance, with the AUC increasing from approximately 0.70 to 0.78–0.80. River network density emerged as the most important single predictor, followed by forest cover ratio and temperature lagged by two months. This suggests that combining surveillance data with routinely available climatic, hydrological, and landscape variables may improve leptospirosis risk stratification at the oblast-month level. A recent systematic review of artificial intelligence applications in infectious-disease early warning systems similarly found that integrating epidemiological, climate, and environmental data sources can improve prediction accuracy across a range of diseases, while also highlighting persistent challenges related to data quality, model transparency, and the limited availability of region-specific training data [33].

Several aspects of these findings should be interpreted in the context of the ongoing war in Ukraine. Occupational exposure among military personnel operating in trenches, wetlands, and other waterlogged environments has been identified as an emerging risk factor for leptospirosis in Ukraine [4], and the war has been associated with broader disruption to infectious-disease surveillance, healthcare access, diagnostic capacity, and data collection [1]. An analysis of open-source epidemic intelligence found that, during the first months of the 2022 invasion, reports of several infectious diseases, including leptospirosis, captured by automated open-source monitoring exceeded those captured by formal surveillance, consistent with reduced testing and reporting capacity in conflict-affected areas [34]. Because several of the lowest-incidence oblasts identified in the present study, including Zaporizhzhia, Kherson, and Kharkiv, are located in regions directly affected by the war, the low reported incidence in these oblasts may reflect, in addition to the ecological factors discussed above, some degree of surveillance disruption, limited access to diagnostic testing, and incomplete data capture, particularly in frontline areas. This would tend to widen, rather than narrow, the apparent gap between high- and low-incidence regions and should be kept in mind when interpreting the magnitude of the regional gradient.

## 5. Limitations

This study has several limitations. First, the analysis was ecological and based on oblast-level aggregated data; therefore, the observed associations cannot be assumed to apply to individual cases or households, and unmeasured regional confounding cannot be excluded. Second, the ecological variables were treated as static characteristics over 2023–2025. Forest cover ratio was based on the 2011 national forest inventory, while river network density captured broad hydrological structure but not seasonal flooding, surface-water dynamics, or wetland extent. More recent and dynamic land-cover and hydrological data could further clarify these associations. In addition, forest cover ratio and river network density were strongly correlated, meaning that their independent contributions cannot be fully separated in this ecological analysis. The 2011 forest inventory may not fully represent land-cover conditions during 2023–2025, particularly because fires, military activity, land abandonment, and other war-related changes may have altered local landscapes. Reliance on MAT may also have resulted in delayed or missed confirmation when paired serum samples or follow-up testing were unavailable.

The exploratory machine-learning analysis was also limited by the relatively small number of region-months. Although adding ecological and climatic-zone variables improved discrimination under standard cross-validation, performance was lower under leave-one-zone-out and leave-one-region-out validation, indicating limited generalization to unseen areas. Finally, reported incidence likely underestimates the true burden because of non-specific early symptoms, reliance on laboratory confirmation methods such as the microscopic agglutination test, and additional diagnostic, reporting, and data-collection challenges associated with the war.

Taken together, these findings suggest that lagged temperature, river network density, and forest cover ratio capture important components of regional leptospirosis risk in Ukraine. Continued monthly oblast-level surveillance combined with simple ecological covariates may provide a practical basis for risk monitoring and for prioritizing areas for further investigation, particularly regarding surface water, wetlands, forested landscapes, and military-related exposure and exposure among displaced populations.

## 6. Conclusions

Leptospirosis incidence in Ukraine during 2023–2025 varied substantially by season and region. The highest rates were recorded in late summer and autumn and in the Carpathian and Polissia climatic zones. Average temperature lagged by two months was the strongest meteorological predictor, while river network density was the leading ecological predictor in the adjusted models. The positive unadjusted association between forest cover and incidence became negative after adjustment for river network density, suggesting that these variables partly captured shared ecological characteristics rather than representing distinct independent effects. The Random Forest classifier showed moderate discrimination under five-fold cross-validation, but its performance was lower when applied to climatic zones and oblasts that were not included in the training data. Monthly epidemiological surveillance at the oblast level, combined with climatic and hydrological data, may support regional risk monitoring. However, war-related disruptions to diagnosis and reporting should be considered when interpreting regional differences.

## Data Availability

The raw data supporting the conclusions of this article will be made available by the authors on request.
